# Sex Differences in Serum Prolactin Levels in Children and Adolescents on Antipsychotics: A Systematic Review and Meta-Analysis

**DOI:** 10.2174/1570159X21666221027143920

**Published:** 2023-05-12

**Authors:** Lidia Ilzarbe, Daniel Ilzarbe, Felipe Gutiérrez-Arango, Inmaculada Baeza

**Affiliations:** 1 Department of Psychiatry and Psychology, Institute of Neuroscience, Hospital Clinic de Barcelona, Barcelona, Spain;; 2 Department of Child and Adolescent Psychiatry and Psychology, Institut Clinic of Neurosciences, Hospital Clínic de Barcelona, IDIBAPS, Barcelona, Spain;; 3 University of Barcelona, Barcelona, Spain;; 4 Centro de Investigación Biomédica en Red de Salud Mental (CIBERSAM), Instituto de Salud Carlos III (ISCIII), Madrid, Spain

**Keywords:** Children, adolescent, prolactin, antipsychotic, sex, meta-analysis

## Abstract

**Background:**

Serum prolactin levels are influenced by sex, physical development and medications among other factors. Antipsychotics usually increase serum prolactin levels in both adults and younger patients, but no study has reviewed the potential association between sex and vulnerability for developing hyperprolactinemia among children and adolescents.

**Objective:**

Systematic review and meta-analysis of serum prolactin levels in children and adolescents on antipsychotic treatment for any psychiatric diagnosis to determine the effect of sex.

**Methods:**

A systematic search was performed in MEDLINE/PubMed/Web of Science and Cochrane databases for randomized controlled trials of antipsychotics in children and adolescents reporting serum prolactin levels by sex.

**Results:**

Of 1278 identified records, seven studies were included, comparing different single antipsychotics to placebo (risperidone N=4; lurasidone N=1; olanzapine N=1; queriapine N=1). Both male and female children and adolescents on antipsychotics presented a significant increase in prolactin levels relative to subjects receiving a placebo. (Male: 16.53 with 95% CI: 6.15-26.92; Female: 26.97 with 95% CI: 9.18-44.75). The four studies using risperidone had similar findings (Male: 26.49 with 95% CI: 17.55-35.43; Female: 37.72 with 95% CI: 9.41-66.03). In the direct comparison between sexes, females showed greater increases in prolactin, but the differences were not statistically significant.

**Conclusion:**

Serum prolactin levels are increased in children and adolescents of both sexes on antipsychotics, with females showing a slightly greater increase than males. Further research is needed to clarify the influence of sex and pubertal status on prolactin levels in children and adolescents taking antipsychotics.

## 1 INTRODUCTION

Prolactin (PRL) is a polypeptide secreted by the anterior pituitary gland, whose main known action is its lactotrophic function [1, 2]. The release of this hormone comprises two different modes: one pulsatile (burst-like) and one basal (time-invariant), and it is conditioned by many different factors, including hormone regulation, sex, age, and medications [1].

Regarding hormone regulation, it has been widely described that dopamine has an inhibitory action on PRL secretion, which is mediated by its binding to type-2 dopamine (D2) receptors located on the membrane of lactotrophic cells [1-3]. This binding activates a wide range of intracellular signaling that modifies gene expression and leads to a decrease in PRL exocytosis. At the same time, PRL has an auto-feedback mechanism for regulating its own release [1, 3]. Additionally, a variety of other molecules indirectly modulate PRL secretion through their effects on the dopaminergic system, disinhibiting (*e.g.,* serotonin, GABA, oestrogens and opioids) or reinforcing it (*e.g.,* substance P). The physiological role of various releasing hormones has been found to vary between male and female subjects, resulting in sex differences in PRL secretion [3].

The difference between males and females in serum PRL levels has been broadly reported in scientific literature, and it seems especially notable in both postmenopausal women and females in the perinatal period [1, 4, 5]. PRL levels during and after menopause tend to be lower than during the fertile period of women. Similarly, PRL levels increase in the perinatal period, suggesting a possible effect of oestrogens on PRL secretion. Taking this into account, most studies have established different PRL values for male and female subjects for defining hyperprolactinemia. This condition consists of a sustained increase in serum PRL levels unrelated to specific physiological functions (*e.g.* pregnancy, lactation) [5, 6]. Concentrations above the upper normal PRL limit have been associated with significant health problems, including gynecomastia, galactorrhoea, breast cancer, sexual dysfunction, alterations in reproductive function and the menstrual period, ostheoprosis, and metabolic alterations [6, 7].

In addition to sex, age has been described as a regulating factor of PRL release. Thus children and adolescents are considered an at-risk population for presenting elevated serum PRL levels [2, 4, 8]. Studies with healthy children and adolescent populations suggest an effect of age and sexual maturation measured with the Tanner Stage. Tanner Staging, also known as Sexual Maturity Rating (SMR), is an objective classification system that measures secondary sex characteristics during puberty, classifying it into five stages [9]. Elevated serum PRL levels have been found at birth and at the onset of puberty, especially for females [10, 11]. A higher density of D2 receptors in the central nervous system of young individuals could explain this difference. It is possible that AP would occupy the D2 receptors and thus prevent dopamine from exerting its inhibitory effect on PRL secretion [7].

The child and adolescent population is especially vulnerable when other factors that may stimulate PRL secretion are present. These include pharmacological treatments such as antipsychotic (AP) agents. In this regard, hyperprolactinemia is present in up to 70% of patients undergoing AP treatment [2, 5]. The suggested mechanism for explaining this increase in serum PRL levels is that AP agents may block D2 receptors in the pituitary gland by crossing the blood-brain barrier, thus inhibiting the dopaminergic system and permitting PRL release [6, 7]. The increase in serum PRL levels caused by AP has a dose-dependent effect [6], appearing from the beginning of AP treatment and tending to stabilize with long-term AP treatment [6, 12]. The AP agents with the greatest risk of inducing higher serum PRL levels are first-generation antipsychotics and certain second-generation antipsychotics (risperidone, paliperidone, amisulpride and sulpiride) [5, 6, 13].

Recent meta-analyses have reported data on the efficacy and safety of AP treatment on children and adolescents [14-16], but only one has focused on serum PRL levels [5]. That study looked specifically at children and adolescents with schizophrenia. Interestingly, as in previous studies with adults [4, 6, 7, 17, 18], the authors found higher PRL concentrations in children and adolescents upon AP treatment compared to placebo. Both males and females experienced this increase, with females showing slightly higher PRL levels. To our knowledge, no published studies have directly analyzed the effect of sex in the child and adolescent population as a factor that may affect the risk of developing hyperprolactinemia. The main purpose of the present study is to systematically review and meta-analyse serum PRL levels in children and adolescents following AP treatment and the effect of sex.

## 2 MATERIAL AND METHODS

### 2.1 Search Strategy

This study was conducted in accordance with the Preferred Reporting Items for Systematic Reviews and Meta-Analyses (PRISMA) guidelines [19]. The protocol of this study was registered on the International Prospective Register of Systematic Reviews (PROSPERO) (ID: CRD42022277202).

A systematic search was performed in MEDLINE/PubMed/Web of Science and Cochrane databases for indexed records from inception until 25^th^ October 2021, using the following search terms: (Adolescent OR Adolescents OR Adolescence OR Youth OR Young OR Teen OR Teenager OR Child OR Children) AND (Amisulpride OR Aripiprazole OR Asenapine OR Benperidol OR Brexpiprazole OR Cariprazine OR Chlorpromazine OR Clopenthixol OR Clozapine OR Flupenthixol OR Fluphenazine OR Fluspirilene OR Haloperidol OR Iloperidone OR Levomepromazine OR Loxapine OR Lurasidone OR Molindone OR Olanzapine OR Paliperidone OR Quetiapine OR Penfluridol OR Perazine OR Perphenazine OR Pimozide OR Risperidone OR Sertindole OR Sulpiride OR Sultopride OR Thioridazine OR Thiothixene OR Trifluoperazine OR Ziprasidone OR Zotepine OR Zuclopenthixol OR Neuroleptic OR Antipsychotic) AND (Prolactin OR PRL OR HPRL OR Hyperprolactinemia OR Hyperprolactinaemia). Furthermore, reference lists of relevant studies were scanned to identify additional eligible reports. No limitations on language, type of document or publication status were applied to the search.

### 2.2 Selection Criteria

The Population, Intervention, Comparison, Outcome (PICO) strategy was used to define inclusion criteria. Population: children and adolescents (mean age of samples ≤18 years), including males and females, with any diagnosis or symptom, including healthy volunteers. Intervention: antipsychotic treatment with any route of administration. Comparison: antipsychotic treatment group compared to a placebo group. Outcome: reported changes in serum PRL levels stratified by sex. Study design: double-blind or open-label, placebo randomized controlled trial (RCTs). Observational studies, conference abstracts, letters, theoretical articles, reviews and books were excluded.

Additional exclusion criteria were as follows: a) trials encompassing samples with hyperprolactinemia at baseline or with a diagnosis affecting the hypothalamus-pituitary-adrenal axis (*e.g.*, hypogonadism, growth hormone deficiency); b) studies comparing potentiation strategies of AP treatment or non-commercialized drugs (*e.g.*, n-metil-amisulpride, JNJ-37822681); c) trials whose duration of AP treatment was <7 days, or allowing concomitant medications or drugs that had not been stable over the 30 days prior to the study, or permitting cross-tapering strategies at the beginning of the trial, due to its potential influence on PRL levels.

### 2.3 Selection of Studies

The selection process was conducted in three separate steps. First, duplicated studies were identified by using an automation tool (Rayyan; available at: https://www.rayyan.ai/) and then revised by two independent authors (LI and FG-A) to exclude them. Second, the titles and abstracts of all non-duplicate articles were independently screened by the two researchers, excluding trials not meeting inclusion criteria. Finally, the two researchers assessed the full-text version of all remaining articles for eligibility, according to the inclusion criteria previously defined. Discrepancies were resolved by discussion among all co-authors.

### 2.4 Quality Assessment

The methodological quality of the selected articles was assessed following the Evidence Project risk of bias tool [20] and the Jadad scale [21]. The Evidence Project risk of bias tool is used for evaluating intervention studies and consists of a simple checklist with no summary judgment. This tool examines eight items for measuring aspects related to comparison strategy, randomization, and description of loss at follow-up. The Jadad scale evaluates methodological characteristics of RCTs, including a total of 3 items regarding randomization, masking and description of loss at follow-up. From these items, a summary judgment is obtained. Studies were classified as low quality when the Jadad score was lower than 3; while a Jadad score of 3 or higher indicated high quality.

### 2.5 Data Extraction

The following data were extracted from each study, using a purpose-built data extraction spreadsheet: first author, year of publication, study design, trial period, diagnosis, diagnostic criteria, sample size, demographic characteristics of participants (age, sex, and race), an antipsychotic treatment used in the intervention group, mean dose of antipsychotic and mean change and standard deviation (SD) in serum PRL levels stratified by sex in both groups (intervention and placebo group). The mean doses of prescribed AP were converted to chlorpromazine equivalents [22].

### 2.6 Statistical Analyses

A meta-analysis was conducted in R statistics version 3.6.3, using library *metaphor* for V.24-0. Summary estimates and their standard deviations were calculated using a random-effects model, considering the between-study variability. Statistical heterogeneity was evaluated with Cochran’s Q test; risk of bias was assessed with funnel plots and Egger’s test. A *p*-value < 0.05 was considered statistically significant.

## 3 RESULTS

### 3.1 Search Strategy

A total of 1278 records were identified through the search strategy and screened by title or abstract, after 51 duplicates were removed. Of them, 150 studies were included for full-text assessment, after which 7 RCTs were included in the present meta-analysis [23-29]. The reasons for excluding the remaining 143 records are listed in Fig. (**1**).

### 3.2 Characteristics of Included Studies

All of the 7 studies included in the meta-analysis were double-blind RCTs comparing placebo *versus* risperidone (n=4, [23, 26-28]), lurasidone (n=1, [25]), quetiapine (n=1, [24]) and olanzapine (n=1, [29]), with a mean dose of AP of 393.1 mg/day equivalent dose of chlorpromazine. There were no reported differences in the doses received of AP treatments between males and females in any of the studies. The follow-up period was 6 weeks for most of the studies (n=5, [23-25, 27, 28]), while in two RCTs, it was only 3 weeks [26, 29]. Taken together, the studies included 1147 patients, with the following main diagnoses: schizophrenia (n=3, [24, 25, 27]), conduct disorder, oppositional defiant disorder or disruptive behaviour disorder not otherwise specified (n=2, [23, 28]) and manic or mixed bipolar episodes (n=2, [26, 29]). Diagnosis was based on Diagnostic and Statistical Manual of Mental Disorders (DSM), Fourth Edition (DSM-IV) (n=4, [23, 26-28]) and DSM-IV, Text-Revised (DSM-IV-TR) (n=3, [24, 25, 29]), with 4 studies adding the Kiddie-Schedule for Affective Disorders & Schizophrenia, Present & Lifetime Version (K-SADS-PL) criteria [24-27]. A summary of the characteristics of included studies is presented in Table **1**. The total number of participants whose serum PRL levels were measured was 425 in the placebo groups (39.3% of females) and 722 in the intervention groups (38.9% of females). Mean ages were 13.07 years (range: 8.1-15.5) in the placebo group *versus* 13.16 (range: 8.6-15.7) in the intervention groups of the included studies. A complete description of the characteristics of participants and PRL values is recorded in Table **2**.

### 3.3 Quality of Included RCTs

The Evidence Project risk of bias tool found a good study design in all of the included trials. Moreover, all studies found an adequate equivalence of comparison groups except one that did not report equivalence outcome measures between groups. Regarding participant representativeness, none of the studies performed a random selection of participants for assessment, and three of them presented a follow-up rate ≥ 80%. More details are described in Supplementary Table **S1**.

Six out of seven studies were designated as high-quality studies according to the Jadad scale, two with a maximum score of 5 and four with a score of 3. A complete description of the scores obtained on the Jadad scale for each study is presented in Supplementary Table **S2**.

### 3.4 Results of the Meta-Analyses

**Fig. (2):**
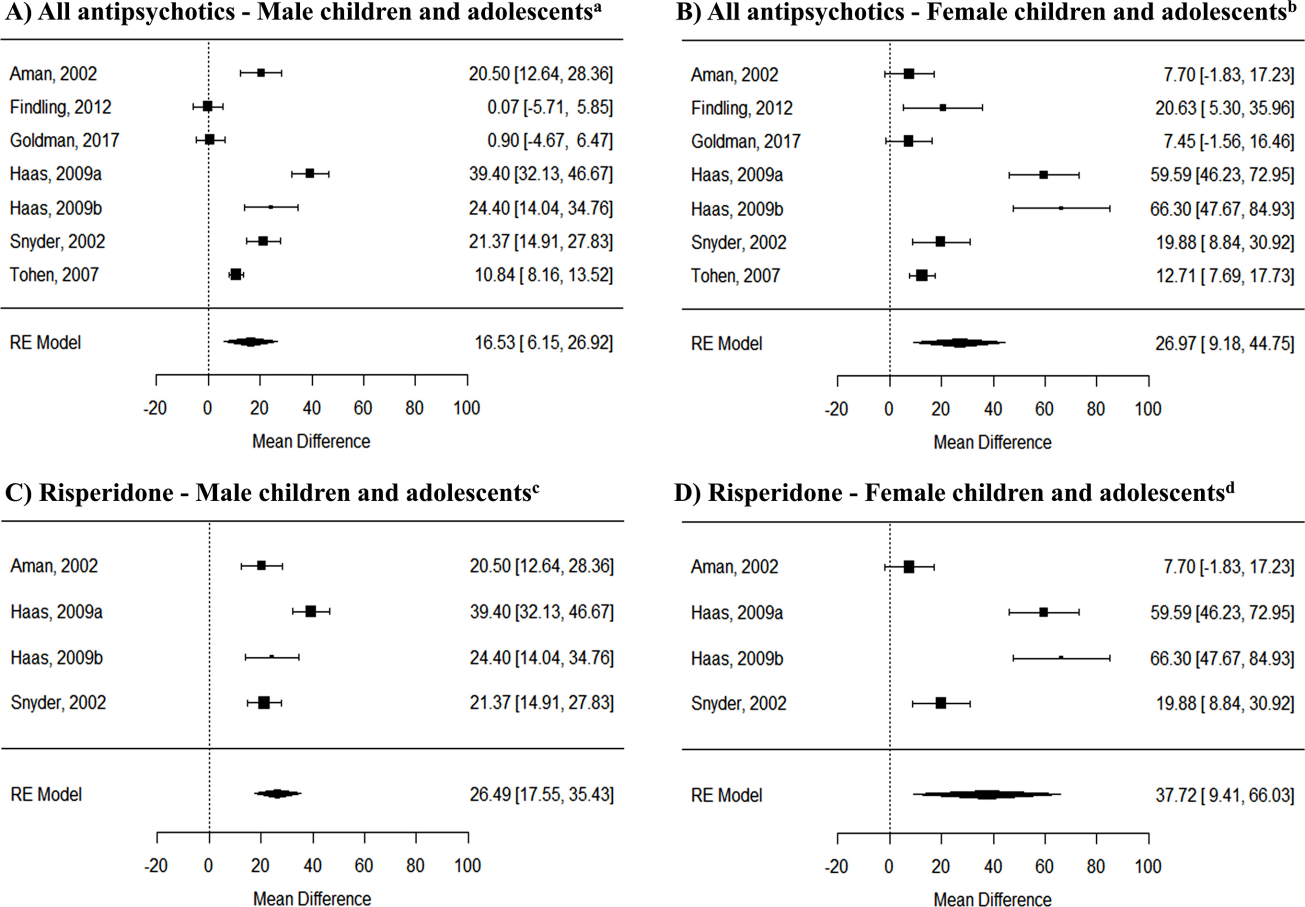
Forest plot of randomized control trials of all antipsychotics prescribed to children and adolescents evaluating prolactin changes by sex, in males (**A**) and females (**B**); and of randomized control trials of risperidone prescribed to children and adolescents evaluating prolactin changes by sex, in males (**C**) and females (**D**) relative to placebo.

Both the male and female groups of children and adolescents undergoing treatment with an AP presented a significant increase of PRL levels relative to those receiving placebo (Male: 16.53 ng/ml with 95% CI: 6.15-26.92 ng/ml, p = 0.0018; Female: 26.97 ng/ml with 95% CI: 9.18-44.75 ng/ml, p = 0.003). Regarding treatment with risperidone, again both male and female children and adolescents receiving this treatment presented a significant increase of PRL levels relative to those receiving placebo (Male: 26.49 ng/ml with 95% CI: 17.55-35.43 ng/ml, *p* < 0.0001; Female: 37.72 ng/ml with 95% CI: 9.41-66.03 ng/ml, *p* = 0.009) (Fig. **2**).

A secondary meta-analysis was performed to directly compare PRL levels between males and females. There were no statistically significant differences in the increase of PRL levels between male and female children and adolescents currently taking any AP (mean change difference: 5.16 ng/ml with 95% CI: -6.22-16.53 ng/ml, *p* = 0.37). There were also no statistically significant differences between the sexes, looking specifically at risperidone (mean change difference: 9.96 ng/ml with 95% CI: -11.77-31.70 ng/ml, p = 0.37) (Supplemental Fig. **S1**).

There was no significant asymmetry between the studies included in each meta-analysis performed, as shown by Egger’s tests, and Begg’s funnel plots, except for the secondary meta-analysis between sex differences in RCTs using risperidone (Egger's test for funnel plot asymmetry: t = 4.97, df = 2, p = 0.04) (Supplemental Figs. **S1** and **S2**).

## 4 DISCUSSION

The increase in PRL levels among patients receiving AP treatment has been broadly reported in previous scientific literature, especially in adult populations [4, 7, 17, 18]. Although still scarce, studies performed in child and adolescent populations have replicated these results, finding higher serum PRL levels in subjects treated with AP agents compared to placebo groups [29-31]. To our knowledge, there is only one previous meta-analysis [5] investigating changes in serum PRL levels in children and adolescents, and this showed higher PRL concentrations in patients with schizophrenia receiving treatment with AP compared to placebo. That meta-analysis also looked at sex differences, focusing specifically on studies recording PRL levels separately for each sex (3 of 5 selected studies). The study found that both males and females upon AP had statistically significant increases in PRL concentrations compared to their respective placebo groups, but the authors did not directly compare males *versus* females. In our review, two [24, 27] of these three studies analyzed by Balijepalli *et al.* [5] have also been selected, while the third one [32] was excluded because of the use of concomitant treatments during the trial (*e.g.,* ziprasidone, quetiapine, haloperidol, levomepromazine, zuclopenthixol) which may have modified serum PRL levels. Our findings, in what was a larger meta-analysis (7 studies) with a wider range of diagnoses (*e.g.,* not only schizophrenia) are in line with their results. We found that females showed somewhat greater increases of prolactin compared to males, but these differences were not statistically significant.

Our findings show that there is an increase in PRL levels in both male and female children and adolescents during AP treatment and point to differences between sexes that warrant further study. Previous literature evaluating the impact of AP treatment on PRL levels in children and adolescents have reported contradictory results: no change [25, 33], changes only in females [34], changes only in males [23], or changes in both sexes [29]. Inconsistent data regarding sex differences in serum PRL levels could be due to the influence of several factors. First, the roles of different physiological hormones that may influence the secretion of PRL. Estrogens have been suggested to increase PRL release by enhancing the number of lactotrophic cells in the anterior pituitary gland and decreasing dopamine concentration in the hypothalamus [6]. Concentrations of estrogens vary throughout life for both males and females, and this may explain sex differences in serum PRL levels observed between the sexes and in different age groups. The hypothalamic-pituitary-gonadal axis is transiently activated at birth, and estradiol achieves its maximal levels during the first two to four months of life [35]. Interestingly, newborns show high serum PRL levels that remain during the first year of life [10, 36, 37]. During childhood, the hypothalamic-pituitary-gonadal axis enters a quiescent state leading to a decrease of serum estradiol, and although bioactive estrogens are still present, they have little physiological relevance. Estradiol levels become high again in early puberty in females and in late puberty in males, which is generally when the first differences between sexes appear [35]. Similarly, during the pubertal period studies have found higher serum PRL levels in females compared to males [10, 11, 37, 38]. As women approach menopause, estrogen levels decrease, which may, once again, relate to the fact that postmenopausal women show lower serum PRL levels compared to premenopausal women [1, 6]. Similarly, higher serum PRL levels have been found in elderly males, in which the levels of bioavailable estradiol are increased [1]. These variations of serum PRL levels, which may be influenced by estrogens concentrations, might also explain the differences in sensitivity to AP induced hyperprolactinemia between sexes found across the lifespan.

Secondly, the previous use of AP medications should be taken into account due to a kind of tolerability phenomenon where the increase in PRL serum concentrations related to AP treatment appears to attenuate over time. In long-term studies in children and adolescents following AP treatment, an eventual decrease in serum PRL levels has been observed, with levels reaching near-normal values [39, 40], although differences between males and females have not been analysed. In the TEOSS study's acute phase, an 8-week, double-blind RCT, significantly elevated PRL levels in participants taking risperidone compared to molindone and olanzapine were reported [41]. An extension study was conducted [40] with these same children and adolescents (aged 8-19 years) on the same medication for up to 44 additional weeks under double-blind conditions. Interestingly, patients in the risperidone group, when compared with the other treatment groups, showed a significant decrease in serum PRL concentrations at the endpoint [mean decrease from baseline of 11.2±14.1 mcg/L (p=0.004)]. Nevertheless, serum PRL levels remained high in the risperidone group compared to other groups and to their baseline PRL levels at the beginning of the acute-phase study. Significantly, patients receiving risperidone maintained higher PRL levels than other treatment groups throughout the extended follow-up period. This is consistent with Anderson *et al.* [39], who reported that children and adolescents on risperidone treatment had higher serum PRL levels at the endpoint of the acute phase (8 weeks) compared to the values at 22 weeks of follow-up and to the baseline measurements at the start of the trial. The decrease in PRL concentrations described in long-term studies may also be different between males and females, considering the observed differences between both sexes in serum PRL levels during the acute phase of AP treatment in children and adolescents. Nevertheless, there are no studies on this topic and these results should be interpreted with caution, especially when looking at other age groups. A long-term trial of 52 weeks found persistent high serum PRL concentrations in adult patients receiving haloperidol [42]. This may be because PRL secretion depends on a wide variety of conditioning factors as mentioned above, that may cause a reduction of serum PRL concentrations, particularly in child and adolescent populations.

Third, the type of AP drug may also interfere with the results concerning sex differences due to the non-identical potential effect of each AP agent for increased serum PRL levels. Previous studies have shown significant increases in PRL levels in children and adolescents receiving risperidone compared to placebo [31, 43], but also compared to other AP medications such as quetiapine [44, 45], molindone [41], olanzapine [41], and aripiprazole [46]. Olanzapine has also increased serum PRL concentrations in this population compared to a placebo group [29, 30]. A dose-dependent effect has been suggested for various AP agents [27, 33, 40, 43, 47]. Minimal changes in serum PRL levels have been described for some AP such as lurasidone [6, 25], asenapine [48] and quetiapine [6, 44], while others, such as aripiprazole, have been associated with a decrease in PRL concentrations [46, 49, 50].

The diversity of the effect of AP drugs on serum PRL levels has been hypothesized to depend, among other factors, on their binding affinity with the D2 receptors and their capacity to pass through the blood brain barrier [6, 51]. In general, first-generation AP show a lower dissociation constant and a higher affinity and occupancy of D2 receptors, in addition to poorer penetration through the blood-brain barrier compared to second generation AP. Given that the pituitary gland is outside the blood-brain barrier, the poorer capacity of first generation AP to penetrate through this barrier leads to high concentrations of AP in the pituitary gland. The high affinity of first generation AP for D2 receptors results in strong binding and higher occupancy of these receptors, preventing dopamine from binding to D2 receptors. This increases dopamine concentrations in the pituitary gland, which cannot exert its role in blocking PRl release, and results in increased PRL levels in serum [2, 51-53]. In contrast, the vast majority of second-generation AP present a weaker affinity and lower occupancy of D2 receptors, as well as a higher capacity to cross the blood-brain-barrier. As a result, serum PRL levels do not present a significant increase [2, 51-53]. Nonetheless, some second generation AP are associated with increases in serum PRL levels due to their different mechanisms of action, which may involve serotonin receptors and other G protein-coupled receptors (GPCRs), including muscarinic, adrenergic, glutamatergic and histamine receptors, as well as other channels, transporter and enzymes [54, 55]. Specifically risperidone is one of these second-generation AP, linked to higher serum PRL levels [2]. Two of the three selected studies investigating AP other than risperidone, included lurasidone [25] and quetiapine [24] which previous studies have found do not significantly change serum PRL levels. This is worth noting because these studies comprise half of the total sample size of the intervention groups (361 of 722 patients). Of the five remaining studies, one [29] reported results in a population treated with olanzapine, and four with risperidone [23, 26-28]; both AP agents seem to elevate serum PRL levels in children and adolescents as previously described. Nonetheless, two of the RCTs studying risperidone, performed the trial in quite young populations with a mean age of 8.7±2.1 and 8.6±0.3, respectively [23, 28], who were presumably mostly prepuberal. This may be relevant because in addition to the specific characteristics of AP treatment (type of AP and dose-dependent effect), other factors related to human development may affect the findings, and the inclusion of subjects who have not reached the age of sexual maturation could explain the absence of statistically significant differences found between males and females.

Regarding the influence of age on serum PRL levels, the results are inconclusive. As in the present meta-analysis, some studies in healthy children and adolescent populations have reported a tendency towards higher serum PRL concentrations for adolescent females (11-20 years) compared to males, but with no differences found for younger children [10, 11, 37, 38]. In contrast, other studies have found no sex differences in PRL values from 4-19 years [36]. These divergent results may reflect the high individual variability in PRL concentrations, particularly among females. Furthermore, previous investigations not considering Tanner Stages have usually failed to find differences in serum PRL concentrations stratified by sex [11]. This suggests that pubertal status may play a more important role in PRL levels than age in general. Accordingly, Elmlinger *et al.* [10] reported statistically significant differences in PRL concentrations in females at Tanner stages 3, 4 and 5 compared to stage 1, while differences in males were only found in stage 5. In contrast, another investigation [36] showed only slightly higher serum PRL levels in pubertal stage 5 compared to baseline values for both males and females, with differences that did not reach statistical significance.

None of the studies included in our meta-analysis considered pubertal status in their analyses. Only one [27] mentioned the Tanner stages of sexual maturation as one of the characteristics of the participants that had been recorded, but did not include this data in its analysis. It could be helpful for future research to focus on pubertal status when analysing differences between sexes regarding the impact of AP treatment on PRL levels, and their related adverse effects.

Some limitations should be considered when interpreting the results obtained in the present meta-analysis. First, a relatively small number of studies were included. Up to the present, few prospective studies reporting serum PRL levels in children and adolescent populations following AP treatment have been conducted under adequate conditions of blinding and randomization. Even fewer studies have reported results stratified by sex. More research of these specific factors would help establish a clearer picture of the impact of sex on PRL levels in young populations taking AP. A second limitation relates to methodology and specifically the quality of the studies analysed. Two of the included studies were deemed low-quality studies according to the score of the Jadad Scale, receiving the lowest possible score on that scale. More high-quality research in the future would, of course, be helpful to offer a reliable picture of PRL levels in male and female children and adolescents following AP treatment.

## CONCLUSION

This meta-analysis has found a significant increase in serum PRL levels in children and adolescents of both sexes following AP treatment. Levels were generally higher in females than males, but these differences did not reach statistical significance. Further research is needed to offer a clearer picture of the influence of sex, pubertal status and AP treatment on PRL secretion and serum levels. Future clinical trials with high-quality methodology (*e.g.,* prospective RCTs in adequate conditions of blinding and randomization, controlling concomitant medications which may affect PRL levels) should systematically report PRL levels for males and females separately while also reporting not only the age but the pubertal status of the subjects.

## Figures and Tables

**Fig. (1):**
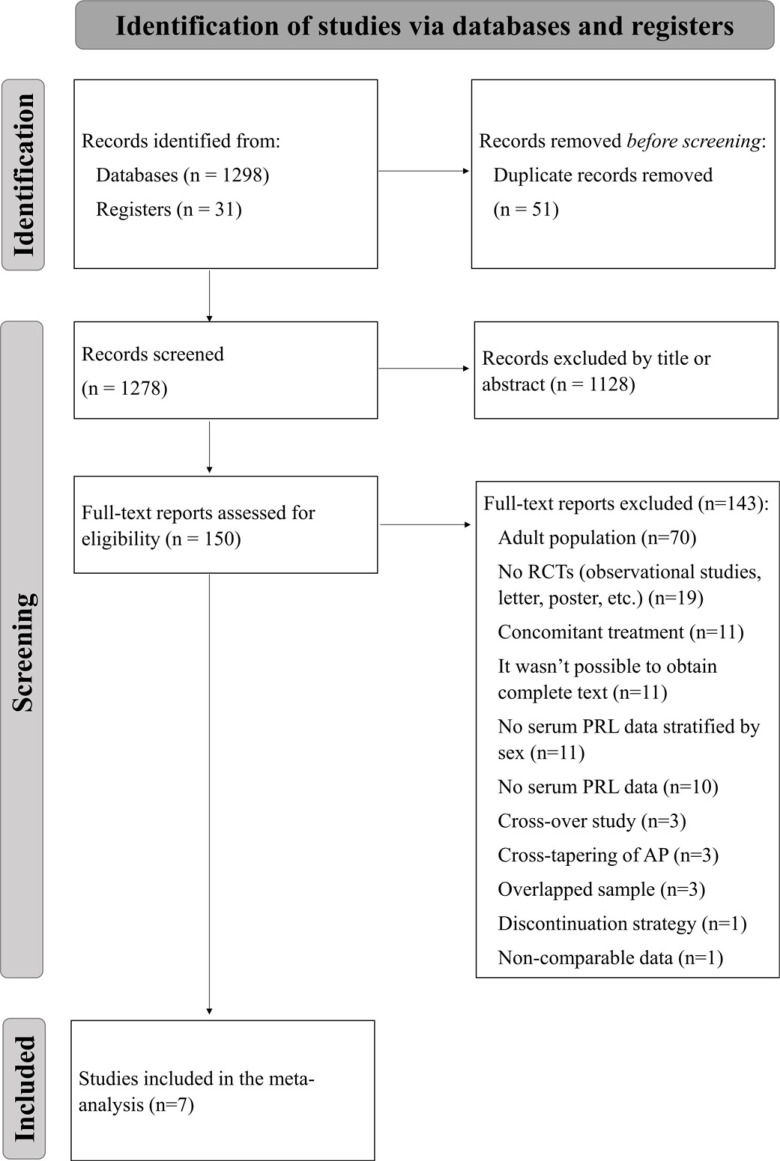
PRISMA flowchart is reporting the search strategy of the meta-analysis [19].

**Table 1:** Characteristics of included studies in the meta-analysis.

**References**	**Design**	**Trial Period (Weeks)**	**Assessment of Prolactin Levels (Weeks)**	**Diagnosis**	**Diagnosis ** **Criteria**	**Inclusion Criteria**
Aman *et al.*, 2002 [23]	DB-RCT	6	6	CD, ODD, or DBD-NOS	DSM-IV	Aged 5-12 years; CP-NCBRF ≥24; IQ 36-84
Findling *et al.*, 2012 [24]	DB-RCT	6	6	Schizophrenia	DSM-IV-TR + K-SADS-PL	Aged 13-17 years; PANSS ≥60
Goldman *et al.*, 2017 [25]	DB-RCT	6	6	Schizophrenia	DSM-IV-TR + K-SADS-PL	Aged 13-17 years; PANSS ≥70; CGI-S ≥4
Haas *et al.*, 2009^a^ [26]	DB-RCT	3	3	Manic or mixed bipolar episodes (BD-I)	DSM-IV + K-SADS-PL	Aged 10-17 years; YMRS ≥20
Haas *et al.*, 2009^b^ [27]	DB-RCT	6	6	Schizophrenia	DSM-IV + K-SADS-PL	Aged 13-17 years; PANSS 60-120
Snyder *et al.*, 2002 [28]	DB-RCT	6	6	CD, ODD, or DBD-NOS	DSM-IV	Aged 5-12 years; CP-NCBRF ≥24; IQ 36-84
Tohen *et al.*, 2007 [29]	DB-RCT	3	3	Manic or mixed bipolar episodes	DSM-IV-TR	Aged 13-17 years; YMRS ≥20

**Abbreviations**: DB-RCT=double-blind randomized controlled trial; CD=conduct disorder; ODD=oppositional defiant disorder; DBD-NOS=disruptive behaviour disorder not otherwise specified; BD-I=Bipolar Disorder type I; DSM: Diagnostic and Statistical Manual; TR: text revision; K-SADS-PL=Kiddie-Schedule for Affective Disorders & Schizophrenia, Present & Lifetime Version; CP-NCBRF=Conduct Problem subscale of the Nisonger Child Behavior Rating Form; IQ=Intelligence Quotient; PANSS= Positive and Negative Syndrome Scale; YMRS=Young Mania Rating Scale; CGI-S= Clinical Global Impressions-Severity.

**Table 2:** Characteristics and prolactin data of participants of the included trials.

**References**	**Treatment**	**Dose (mg), ** **Mean ± SD**	**N**	**Age (Years), ** **Mean ± SD**	**Caucasian, ** **N (%)**	**PRL ** **Assessment, ** **N (%)**	**Male, ** **n (%)**	**PRL Change at Follow-Up, Males (ng/ml), Mean ± SD**	**Female, ** **n (%)**	**PRL Change at Follow-Up, Females (ng/ml), Mean ± SD**
Aman *et al.*, 2002 [23]	Placebo	-	63	8.1 ± 2.3	19 (30.2)	53 (84.1)	40 (75.5)	1.8 ± 7.6	13 (24.5)	0.7 ± 3.5
	Risperidone	1.2 ± 0.6	55	8.7 ± 2.1	21 (38.2)	41 (74.5)	34 (82.9)	22.3 ± 22.3*	7 (17.1)	8.4 ± 12.6
Findling *et al.*, 2012 [24]	Placebo	-	73	15.34 ± 1.4	46 (63.0)	63 (86.3)	36 (57.1)	-6.5 ± 15.1	27 (42.9)	-33.9 ± 34.9
	Quetiapine	400	73	15.45 ± 1.3	45 (61.6)	63 (86.3)	37 (58.7)	-9.2 ± 14.4	26 (41.3)	-12.4 ± 18.5
	Quetiapine	800	74	15.45 ± 1.3	44 (59.5)	60 (81.1)	36 (60.0)	-3.7 ± 11.6	24 (40.0)	-14.04 ± 39.1
Goldman *et al.*, 2017 [25]	Placebo	-	112	15.3 ± 1.4	74 (66.1)	112 (100)	71 (63.4)	0.1 ± 22	41 (36.6)	-2.3 ± 22.8
	Lurasidone	40	108	15.5 ± 1.3	72 (66.7)	110 (101.9)	68 (61.8)	-0.1 ± 11.1	42 (38.2)	2.4 ± 35.3
	Lurasidone	80	106	15.3 ± 1.4	74 (69.8)	104 (98.1)	69 (66.3)	2.1 ± 15	35 (33.7)	7.9 ± 14.4
Haas *et al.*, 2009^a^ [26]	Placebo	-	58	13^c^	45 (77.6)	54 (100)	26 (49.1)	0.6 ± 6.7	27 (50.9)	1.6 ± 7
	Risperidone	NR (range: 0.5-2.5)	50	13^c^	35 (70.0)	55 (100)	24 (53.3)	32 ± 22.5	21 (46.7)	50 ± 45.6
	Risperidone	NR (range: 3-6)	61	13^c^	50 (82.0)	51 (100)	20 (37.7)	49.6 ± 23	33 (62.3)	68.3 ± 49.1
Haas *et al.*, 2009^b^ [27]	Placebo	-	54	15.4 ± 1.4	27 (50.0)	36 (63.2)	35 (64.8)	-3.2 ± 24.8	19 (35.2)	-9.2 ± 24.1
	Risperidone	NR (range: 1-3)	55	15.5 ± 1.3	33 (60.0)	33 (62.3)	30 (54.5)	16 ± 23.7	25 (45.5)	36.9 ± 41.3
	Risperidone	NR (range: 4-6)	51	15.7 ± 1.3	24 (47.1)	54 (100)	37 (72.5)	26.4 ± 28.5	24 (47.1)	77.3 ± 60.8
Snyder *et al.*, 2002 [28]	Placebo	-	57	8.8 ± 0.3	42 (73.7)	107 (100)	26 (72.2)	-1.25 ± 3.8	10 (27.8)	-1 ± 2.2
	Risperidone	1 ± 0.1	53	8.6 ± 0.3	41 (77.4)	53 (84.1)	25 (75.8)	20.1 ± 16.1*	8 (24.2)	18.88 ± 5.2*
Tohen *et al.*, 2007 [29]	Placebo	-	54	15.4 ± 1.2	41 (75.9)	41 (74.5)	24 (44.4)	0.7 ± 3.1	30 (55.6)	2.67 ± 8.6
	Olanzapine	8.9 ± NR	107	15.1 ± 1.3	71 (66.4)	63 (86.3)	61 (57.0)	11.5 ± 9.5*	46 (43.0)	15.38 ± 13.7*

**Abbreviations**: SD=standard deviation; PRL=prolactin; NR=not reported; ^c^=median age; *p < 0.05.

**Note**: Haas *et al.*, 2009^a^ [26] and 2009^b^ [27] found a dose-dependent increase with risperidone in mean prolactin levels and mean changes higher in females than in males.

## LIST OF ABBREVIATIONS

AP: Antipsychotic
GPCRs: G Protein-coupled Receptors
PRISMA: Preferred Reporting Items for Systematic Reviews and Meta-Analyses
PRL: Prolactin
RCTs: Randomized Controlled Trial
SD: Standard Deviation
SMR: Sexual Maturity Rating

## SUPPLEMENTARY MATERIAL

PRISMA checklist is available as supplementary material on the publisher’s website along with the published article.
